# The Growth of Hospital Literature

**Published:** 1914-10-10

**Authors:** 


### THE GROWTH OF HOSPITAL LITERATURE.

### The New Tendency and its Significance.

With the commencement of a new volume we have cast a glance backward, as our custom is, over the various departments of work whose development is recorded in our columns, and one of the features which presents itself most remarkably is the growth of hospital literature. It is important, however, not merely to move with the times but to become conscious of their tendency, and as regards books about hospitals that tendency is this. Workers in hospitals, whether paid or voluntary, and all those actively interested in their support and management are attaching every year greater importance to hospital literature. There is a marked determination to train the paid and voluntary workers in every department, and this has shown itself in the growth of publications dealing with every branch of the work with a minuteness and consistency that would have seemed impossible a few years ago. One primary cause of this is perhaps hardly realised.

It is that the action of King Edward in founding the great fund associated with his name has caused many of the most capable and trusted men of business both in London and in the great provincial centres to become associated with hospital management. In some large firms, indeed, it is the custom for every partner to become actively associated in the management of at least one hospital, with the result that not only have the institutions gained by receiving the voluntary gift of the best brains and energies, but the leisure of the business men themselves has produced a permanent and growing preoccupation with a vast subject around which a literature of its own is growing up. Parallel with this tendency we find the technical training of the paid officials in every branch of their work necessitating the publication of a series of books and manuals specially written to crystallise existing knowledge on their subject, and to heighten their interest in their work. Medical students, young doctors, nurses, all now possess a literature of their own, and committee-men will soon follow suit. The technical manual, the hospital history, works on construction and design, even plays and novels dealing with the hospital, the country practitioner, the nursing home, and the life of institutional workers, all are now regular features of every publishing season. The medical essayist, giving, significantly, opinions on things from the point of view of Harley Street, is also much in evidence. Busy hospital superintendents and secretaries turn the fragments of their leisure to account by writing histories, by reading papers, contributing special articles, and delivering addresses, and the older and more honoured superintendents and secretaries are earning a well-deserved fame in training the younger members of their staffs to occupy the higher positions. Indeed, each secretary's office in the best administered hospitals is developing into a school of administration; and there is competition nowadays among junior men not merely for posts as such, but for a training under So-and-so, and in the methods of such-and-such a hospital administrator.

This means in practice that there is a thirst for knowledge, which seeks satisfaction in the number of books now published in increasing quantity on every side and branch of hospital work. Our own efforts to stimulate and encourage this interest in fuller measure every year stand recorded in the far wider range of volumes reviewed in our columns of late than was the case a few years ago. The "Institutional Library" section of The Hospital, in the first issue of every month, shows how we have tried to meet this tendency by noticing all new books, pamphlets, and papers on hospital subjects as issued in these pages devoted to reviews. During the past twelve months the lives of Lister and Florence Nightingale, works on first-aid and bandaging; treatises on school clinics; books about babies; radium therapeutics and massage; the histories of St. George's and Bethlem Hospitals; and some interesting historical researches into the hospital movement in the eighteenth century; medical travel, and writers on remote countries like Kashmir and Uganda; books about radium; remarkable biographies like that of Dr. Joseph Bell, the prototype of Sherlock Holmes; books on medical electricity and its department; important works on sanatoria, like the new edition of Dr. F. R. Walker's well-known volume; new medical dictionaries; pamphlets on insurance questions, and the Mental Deficiency Act; these, together with inevitable volumes of purely medical interest and on pharmacy questions form the first rush of bubbles on the literary stream. School design and school treatment, mental diseases and after-care generally, a history of ambulance work; personal studies like the story of Helen Keller, the blind deaf-mute who became one of the most cultivated products of America; such psychological volumes as Mr. A. F. Shand's "Foundations of Character," reviewed under the title of the "path to promotion"; these taken at random indicate the immense field. People now want, significant change, not merely technical volumes dealing with the minutiæ of their profession, though of these we certainly have no lack, but also books on general subjects written from their professional point of view. Harley Street, as we saw, has become an essayist, town and country practice has its well-observed novels, and medical men like Mr. F. G. Layton and grand-masters of European literature like Brieux utilise the material of the consulting-room and even the research laboratory for plays and drama. This change, interesting in itself, has demanded qualities in review writing that were not sought after in old days, and has elevated mere "notices of books" into succinct and lively essays, and made every worker in and supporter of hospitals turn with curiosity and pleasure to what has become a special branch of technical news.

